# Enhancing precision in lymphoma detection with novel deep transformer-based neural networks

**DOI:** 10.1371/journal.pone.0329261

**Published:** 2025-08-13

**Authors:** Turki M. Alanazi

**Affiliations:** Department of Electrical Engineering, College of Engineering, University of Hafr Al Batin, Hafr Al Batin, Saudi Arabia; VIT University, INDIA

## Abstract

Lymphoma appears as swollen lymph nodes and weakened immune-protective tissues, frequently resulting in tiredness and loss of weight. Improving the outlook of this malignancy includes using computer-assisted analysis of Positron Emission Tomography (PET) pictures, which identify changes in metabolism. This article presents an Automatic Pre-Segmentation Model (APSM) that uses the Swin Transformer (ST). The APSM accurately separates inputs by recognizing pixel differences caused by changes in metabolism in various tissues and lymph nodes. Training the Swin Transformer system for classification and identification happens simultaneously, focusing mainly on the lymph node area. The model effectively divides the Lymphoma area by examining differences in patterns between regional features and changes in pixels. This segmentation model combines transformer network training to simultaneously learn fractal variations and feature changes, helping to adjust the relationships between training and testing inputs. The segmentation model’s effectiveness comes from its capability to stop training the matching transformer network when it identifies new deviations, alterations, or both. The proposed model achieved 12.68% higher segmentation accuracy, 13.38% improved precision, and reduced overhead, error, and segmentation time by 12.73%, 9.27%, and 10.23%, respectively, outperforming existing methods.

## 1. Introduction

An uptick in lymphoma cases is observed globally, with 544,352 new instances of non-Hodgkin lymphoma and 83,087 instances of Hodgkin’s lymphoma reported in 2020. High-income nations experienced more cases, while low-income countries had higher mortality rates [1]. Lymphomas are one of the various hematologic cancers. Prognosis and therapy depend on accurately determining the sickness stage and examining histological findings. When treating patients with lymphoma, Positron Emission Tomography (PET) in conjunction with Computed Tomography (CT) is an often-utilized diagnostic modality [2]. Because CT provides detailed anatomic information and PET is sensitive in identifying lymphoma areas, the two methods work well together for better diagnosis and treatment [3]. PET/CT imaging can provide more clarity about the regions of the body that are impacted by lymphoma [4]. Clinical and laboratory parameters used in setting, restaging, and tracking therapy are complemented by the complementary nature of the metabolic and anatomic knowledge obtained from a PET-CT inspection, making it an indispensable part of the treatment of patients [5]. The degree to which PET-CT imaging may prove useful in a given patient’s treatment will depend on the characteristics of that patient’s lymphoma type and its clinical manifestations [6].

A watershed approach with automatic marker control is commonly used to segment a chosen lymph node. The specification of internal and exterior markers is necessary for this method. It was demonstrated that the segmentation task performance depended on the markers chosen [7,8]. Then, the interior and exterior markers are identified using the distorted contour from the baseline scan and the registration of the baseline and follow-up scans [9]. Lymph nodes are frequently surrounded by soft tissues that are difficult to distinguish based only on intensity value. It might be challenging to automatically segment lymph nodes on serial images and consistently separate them from other anatomic structures [10]. The method uses data from baseline images circled by radiologists to provide information about the position of lymph nodes in the images [11]. Utilizing enough structure-level data from the baseline scan enables an informed restriction on the search region for the target lymph node in the new scan, thereby mitigating the surrounding soft tissue difficulty [8,12]. The baseline scan extracts tissue surrounding the lymph node, which is then mapped onto the follow-up scan. Consequently, narrowing down the target lymph node’s search region enhances the outcomes of the automated segmentation process [2,13].

PET/CT analysis using machine learning requires a feature extraction process that converts the image information into a low-dimensional feature vector. The possibility of information loss limits this procedure, which depends on an expert understanding of feature extraction techniques [14]. Therefore, feature design performs less well when working with large and heterogeneous information sets. On the other hand, DL algorithms can immediately analyze data with high dimensions, such as PET/CT images, which gets over the limits brought on by information loss and does away with the requirement for manual feature engineering [15,16]. DL methods perform better in identifying high-volume PET/CT imaging investigations than classic machine learning algorithms due to their practical advantage. CT was recorded with PET images [17]. The CT and PET scan information was combined using a weighted fusion technique. A 3D auto-encoder algorithm also extracts deep learning features (DF) from PET and the fused image. Good performance is obtained using the fusion technique and a suitable automated segmentation technique [16,18]. The main contributions of the paper are:

- An automatic pre-segmentation model using a Swin Transformer detects lymphoma based on pixel distribution and variation.
- Fractal, minimal, and maximum deviation detection from pixel variations and feature changes to effectively improve precision.
- Source-based experimental analysis with different experimental stage descriptions and conditional outputs.
- Comparative study aided by methods, metrics, and variants for the proposed model’s efficiency assessment.

The remaining part of the paper is subdivided into the following sections: Section 2 explains the existing model of identifying lymphoma disease in Related works; Section 3 describes the proposed work APSM with ST; Section 4 reports experimental analysis, whereas Section 5 gives comparative analysis; the conclusion of the study is drawn in Section 6 and the study ends with section 7 suggesting future works.

## 2. Related works

Li et al. [19] developed a leukaemia classification on pet imaging using adaptive assessing and scalable distance regularized level set evolution (AW-SDRLSE). A novel dynamic annular mask determines the average intensity in the surrounding internal and external areas. Ninety cases of actual PET data are used to assess the AW-SDRLSE. The method enhances the lymphoma classification. Wang et al. [20] proposed a Lymphoma Segmentation by Utilising the Spatial-Temporal Correlation. A weighted Dice loss with stable gradient and self-adaptive parameters is used to steady the training procedure. The “UNet” technique creates recurrent dense Siamese decoder architecture. The method maintains extreme categorization effectiveness while reducing the deduction period. Liu et al. [21] introduced a combined lymphoma nodules division and prognosis prediction using multitask convolutional neural networks. The suggested approach shares the picture attributes learned from one task, which helps the learning process of the other task. The approach uses baseline FDG-PET scans to segment lymphoma lesions and predict prognosis. The method uses baseline FDG-PET scans to achieve combined lymphoma nodules division.

As an improved version of [20], Shi et al. [22] suggested a U-Net for automatic lymphoma segmentation in whole-body PET/CT scans. A generative adversarial network (AMC-GAN) is an auxiliary U-Net branch for anatomical-metabolic consistency. Using co-aligned whole-body PET/CT data, AMC-GAN specifically learns representations of normal anatomical and metabolic information. The method enhances the lymphoma classification performance. Zhu et al. [23] evaluated a boundary-optimized system for melanoma localization directed by a cruciform architecture. Semi-automated techniques are paired with human-added information, like limit boxes or tumour position points. The Lymphoma division network (CGBS-Net) is led by a cruciform topology and optimized for boundaries. The approach yields encouraging segmentation outcomes. Regardless of the boundary, an evidence network-based proposal is provided by Huang et al. [24]; who developed a segmenting lymphoma from 3d pet-ct images with a deep evidence network. Semantic feature vectors are extracted from 3D inputs by the feature extraction module using an encoder-decoder structure. The evidence layer computes a belief function at each voxel assessing the unpredictability of the existence. The developed approach enhances the effectiveness of segmentation.

Huang et al. [25] proposed a weakly guided multiple-scale characteristic similarity-based lymphoma separation in PET/CT images. A weakly supervised deep learning approach is used for autonomous lymphoma division to decrease the dependency on precisely labelled datasets. The Atrous Spatial Pyramid Pooling (ASPP) module fuses image features retrieved from several convolutional layers. The suggested approach can lessen the need for expert annotations in lymphoma segmentation. This weakly guided network issues are suppressed using Wang et al. [26] Whole-body lymphoma segmentation based on PET/CT using deep neural networks. Reweighting loss functions are used to train the backbone initially. The prior shift layer uses estimated prognosis reliability to irregularly change prior education information to a more illuminating group. The method enhances the sensitivity and efficacy.

Somaratne et al. [27] suggested a multi-site one-class categorization of follicular lymphoma using generative adversarial networks (GAN). GAN has recently been effectively applied to picture synthesis. The GAN-based method reduced the variations in whole slide images between sizable public data archive sites. The method increases the efficiency. Unlike one-class categorization, Luo et al. [28] evaluated a multi-atlas boundary-aware, context-coordination, UNet-like technique for tumour segmentation. The multi-atlas boundary-aware (MABA) module focuses on unclear regions between tumours and adjacent tissues to acquire probable tumour boundaries. The module is built on a gradient, uncertainty, and level set atlas. The method enhances the accuracy level. Luga et al. [29] developed a thorax lymph node identification and division from CT data. Using this data, fourfold cross-validation was used to train a fully convolutional neural network based on 3D foveal patches. Larger LNs in the instruction set had a higher identification rate than smaller LNs. The method performs excellently overall in the detection process. Blanc-Durand et al. [30] proposed a fully automated division of widespread big B cell lymphoma tumours on 3D FDG-PET/CT. A 5-fold cross-validation approach was employed for instruction with the initial cohort. PET/CTs were used to train a 3D U-net architecture with two input channels for PET and CT. The proposed approach improves the repeatability of TMTV assessments in lymphoma patients.

This proposal is a modified and improved version of [29] proposed by Perry et al. [31], which introduced a deep-learning image-based method for directly detecting high-grade B-cell lymphomas. A brand-new deep learning technology that uses scanned images of biopsy slides to diagnose DHLs and THLs directly. The analysis establishes the viability of using AI to determine DH/TH events. The method enhances the sensitivity and specificity.

Wang et al. [32] suggested combining the extraction of feature maps with the reuse of hierarchical feature maps. A gated convolutional module (GCM) is combined with UNet architecture to form Memory-Net. The cell memories in the GCM form an information highway and the hidden states of GCUs act as feature maps. The method reduces the computational cost. Aghamohammadi et al. [33] evaluated an encoding method for a two-path convolutional neural network for segmenting liver and tumours. Local Direction of Gradient (LDOG) is proposed to illustrate several important aspects within the image. The suggested encoded picture performs exceptionally well in identifying the liver’s border, even in areas near the organs that touch. The method achieves higher precision and effectiveness of segmentation.

Segmentation of lymphoma region from a whole body scan such as PET or CT requires defensive feature extraction as presented in [24,25]. However precise feature maps as in [32] reduce the computational cost through pixel-based selection. Besides, the single layer [27] neural network or dedicated network concepts as in [20,22,29] rely on pixel distribution that improves the segmentation rate. Apart from this, if the distribution detection due to feature variation occurs, then the overhead increases; hence, the layered assessments result in errors. To address such issues from the primary pixel distribution, this article introduces a pre-segmentation model using a Swin Transformer. This paradigm performs independent operations for segmentation and detection, along with variations and changes between different distributions. As the processes are independent, interrupting or mapping less prevents multiple feature assessments. This, in turn, reduces the complexity of handling multi-sized images.

The article introduces an APSM that employs the ST. By accounting for pixel discrepancies brought on by metabolic changes in different organs and lymph nodes, the APSM reliably separates inputs. A simultaneous process of training the Swin Transformer system for recognition and classification occurs, with the lymph node region as the primary emphasis. The model separates the lymphoma area by looking for patterns varying across regions and pixel changes. With transformer network training, this segmentation model can adapt the relationships between testing and training inputs by learning fractal variations and feature changes simultaneously. When the segmentation model detects new deviations, changes, or both, it may cease training the matching transformer network, which is the key to its efficacy.

## 3. Proposed automatic pre-segmentation model using swin transformer

**Fig 1:**
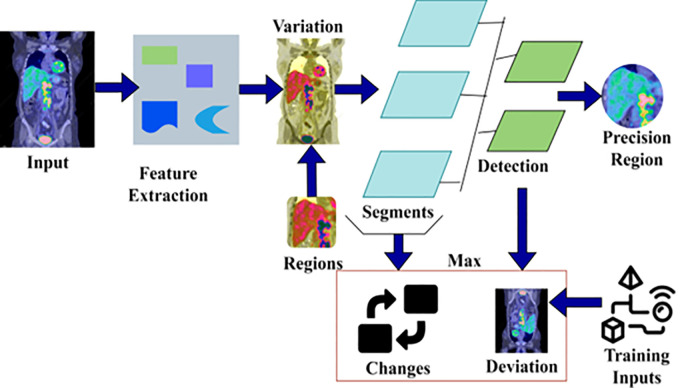
Illustration of the proposed automatic pre-segmentation model.

Lymphoma region segmentation and detection have been pursued to identify and control failed immunity-protective tissues and enlarged lymph node conditions. This identifiable condition leads to weight loss and fatigue. PET image processing output addresses the pixel changes in features and is processed for better cancer-affected region identification. By validating the segmentation and region detection for computing the maximum deviation and changes in pixels based on PET/CT images. The region detection makes it difficult to meet the extracted feature attributes by identifying the lymph node size and fractal deviations used for training the Swin Transformer network. Based on the lymph node size and fractal deviations, the appropriate diagnosis is made to control the growth of cancer cells. Due to metabolism effects across different tissues and lymph nodes, APSM and Swin Transformer models are used to handle the critical situations of lymphoma cancer in the human body. The lymphoma region is segmented independently by computing the accuracy and precision of region features and pixel changes using the Swin Transformer network. The objective of precise lymphoma region identification from the human body with extracted features used for identifying the variations and maximum fractal deviations is consecutively analyzed. In Fig 1 the proposed Automatic Pre-segmentation Model is illustrated.

Different lymphoma-causing conditions can be taken for identifying the precise cancer-affected region from the patients based on pixel changes. In this case, the pixel changes, feature changes, and fractal deviations are trained to adapt the correlation between testing and training inputs. In this scenario, the lymphoma caused by fractal deviations between the pixel changes and region features is to maximize the region detection precision with less overhead and detection time. From the identified regions, the maximum deviation is observed to address complex issues in region detection and variation analysis to reduce false rate occurrence. The continuous monitoring of lymph node size and fractal changes in the patients leads to false rates and improves the segmentation and region detection accuracy. The training of the same transformer network is pursued and terminated from the instance with associated feature attributes and its variations observation. The proposed model operates between fractal deviations and feature changes for maximum region segmentation and detection. In this model, the extracted features attribute and identified variations are used to identify the root cause of lymphoma.

Feature extraction techniques are used to gather information that could show the frequency of lymphoma. This work investigated the efficacy of multistage and multidimensional fractal geometry with colour channels and colour models for lymphoma tissue image classification. Structures that display self-similarity at many scales are the focus of fractal geometry. This idea has applications in medical imaging for analysing lymphoma and other malignancies with complicated forms and textures, which often have fractal-like borders. Fractal analysis at several scales allows for capturing fine and coarse information in the structure. To measure how complicated a structure is fractal dimension. By computing FD at various scales, the irregularities and roughness of lymphoma tumour borders may be captured. This allows for a more thorough description of how the fractal dimensions are distributed throughout the tumour area. It encapsulates the fact that fractal features fluctuate across the tumour.

There are numerous phases to developing an automated pre-segmentation model for lymphoma utilizing PET/CT scans to separate the variations and ROIs within the pictures properly. Magnify PET and CT scans to a significant degree. Ensure the pictures are co-registered so the functional and anatomical data are synchronised. Use thresholding methods to isolate lymphoma-related high-uptake areas in PET scans.

Further, the main goal of handling PET/CT images for identifying the lymph node size and fractal deviation is to reduce the complexity in region segmentation and thereby increase the region detection rate. The APSM uses ST to segment the input images based on the pixel changes, thus reducing failures and lags when segmenting such regions. The ST output is used to classify the maximum deviation and changes identified instances for addressing root causes of cancer by adapting the testing and training inputs continuously. We propose an Automatic pre-segmentation model that joints the outputs of maximum deviations and changes observed from the regions with Swin Transformer.

### 3.1 Feature extraction and discussion

Extracted feature attributes have been analyzed to detect variations in complex regions with high rates under distinguishable input elements. In this feature extraction is performed with input PET/CT images, we assume that the feature belongs to PET images  i, therefore the pixel changes are represented as  (pc=1,2,…,n).

In this analysis, the extracted feature is smaller than the variation detection in such regions. In this scenario, assume that the input segmentation is performed in this model based on pixel changes; it means if the variations do not occur in any region that image is not important to process, and that images show lymphoma disease does not affect that particular patient. Similarly, if the variations addressed in any regions will be terminated and the proper diagnosis recommendation to that patient. First, the variations identified images are processed using Swin Transformer. Different variations observed from the region  r may appear on the segmentation rate $\;{SGM}^{R}$. Each lymph node size and feature changes conditions from the input images contain region segmentation time $\;T^{SGM}$ is validated independently. Based on the analysis, the fractal deviations $\;F^{dv}\left(T^{SGM}\right)$ with feature changes $\;f^{c}(r)$ are continuously computed. In this proposed model, we also consider the maximum deviation in each region. For the analysis, the average detection time ΔT for region r for the first PET image is computed as


$${\Delta T}^{\;r}=\frac{{\Delta T}_{i}}{\left(\left(1-{\left(F^{dv}\left(T^{SGM}\right)\right)}_{r}\right)\left(1-f^{c}(r)\right)\right)}\;if\;1-{\left(F^{dv}\left(T^{SGM}\right)\right)}_{r-1}>V=0$$ (1)

$$=\frac{(pc{)}_{n}*{\Delta T}_{V}}{r\left(F^{dv}\left(T^{SGM}\right)+f^{\;\;c}(r)\right)}\;if\;1-{\left(f^{\;\;c}(r)\right)}_{r-1}\leq V+1$$ (2)

Where


$$\begin{array}{c} {\left(F^{dv}\left(T^{SGM}\right)\right)}_{r}=\sum_{i=1}^{r}\;\rho {\left(C_{x}\right)}_{i},\;with\;V=0 \end{array}$$ (3)

And


$$\rho {\left(C_{x}\right)}_{i}=\frac{{\left({SGM}^{R}\right)}_{i}}{{\left(D^{R}\right)}_{i}}=f^{\;\;c}(r).F^{dv}$$ (4)

**Fig 2:**
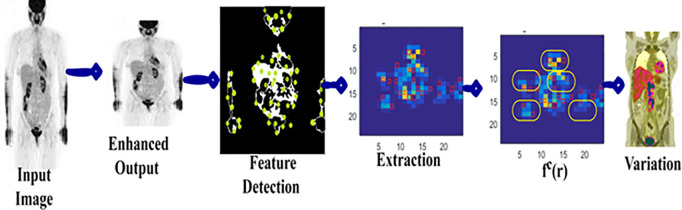
Feature extraction and changes detection.

Where, the variable $D^{R}$ means region detection rate based on the pixel changes  pc to identify the variations  V using the proposed model and Swin Transformer. The probability of addressing complex situations in identifying the feature variations is expressed as $C_{x}$ in any terminated region, the fraction of the next image is processed. Instead, the lymph node size is too big (as high as $\;\rho {\left(C_{x}\right)}_{i}<1$), that images take several minutes to find the pixel changes and fractal deviations in that complex region. The feature extraction process and change detection processes are illustrated in Fig 2.

In the above Fig 2 representation, a sample image from the dataset is used to represent the feature detection and extraction. The extracted features are verified for  V=0 such that $\;\rho \left(C_{x}\right)=0$ under different  r. Therefore $\;f^{c}(r)$ is identified between successive pixels within  r provided $\;D^{R}$ is eased. The $\;SGM^{R}$ is performed if $\;\left[f^{c}(r)\leq V+1\right]$ is satisfied for different  r such that accuracy is improved. The variable ${AV}^{\theta }$ illustrates the average delay observed from the traditional lymphoma image analysis experiences for providing the same diagnosis recommendations or adding additional features to the images and then computing the image processing sequence. Therefore, the average delay is computed as


$$\begin{array}{c} {AV}^{\theta }=\sum_{i=1}^{r}\;\frac{{\left({SGM}^{R}\right)}_{i}\underset{―}{{\left({Sq}^{i}(r)\right)}^{2}}}{V} \end{array}$$ (5)

In equation (5), the variable $\underset{―}{{Sq}^{i}(r)}$ denotes sequential image processing with the segmentation time and detection time is validated. The objective condition is performed to minimize the false rate. The pseudo-code of changes detection in any $\;AV^{\theta }$ is given in Algorithm 1.

Algorithm 1: Changes Detection in  r

$Input:T^{SGM},\;AV^{\theta }$

$Output:f^{c}(r)$

for i=x*y do

define pc={(1,1),(1,2ldots,(i,n)||(n,i)} ∀(i,n)∈(x,y)

V←0

$Compute\;\Delta T^{r}=\frac{p_{c}}{r}$

$While\;\left\{\Delta T^{r}>V\right\}\;do\;loop$

$f^{c}(r)=\left(1+F^{dv}\right)\forall F^{dv}\in r$

$If\left\{F^{dv}\in [0,1]\right\}\;then\;Condition$

$C_{x}\leftarrow pc;V=0\;Update$

$\rho \left(C_{x}\right)=\frac{{\left(SGM^{R}\right)}_{i}}{{\left(D^{R}\right)}_{i}\times F^{Dv}}\;\forall \;T^{SGM}>AV^{\theta }$

End if

$If\;\left\{\Delta T^{r}\leq V+1\right\}then\;condition$

$c_{x}\leftarrow (pc{)}_{i};V=V+1$

$\rho \left(c_{x}\right)=\frac{(pc{)}_{i}}{r}\times \frac{AV^{\theta }}{T^{SGM}}$

for r=1 to i ∀ i∈(x,y)

Repeat from Step 6 Until V=0

End if

$Compute\;f^{c}(r)=\frac{\rho {\left(C_{x}\right)}_{i}}{F^{dv}}$

$Update\;\Delta T^{r}=\frac{(pc{)}_{i}}{r.F^{dv}}$

End for

$Return\;f^{\;\;c}(r)$

### 3.2 Variation detection

The complex problem of identifying lymphoma causes in any region suppresses variations in which segmentation and detection are simultaneously analyzed for precise decision-making done using input images. Each lymph node may differ in size, color, intensity, etc. based on the pixel changes observed from the PET images are further analyzed for providing treatment. The diagnosis recommendation is provided to all the lymphoma-caused patients using Swin Transformer with processing rate ${\;\left(P^{R}\right)}_{i}$. The complex problem in region segmentation and detection process will lead to multiple considerations for handling this disease spread. We assume that segmentation of the affected region from the image is used to compute the maximum deviations from the instance. For this purpose, the ST and final pixel variation output-based changes are observed and compared with the existing model for precise region detection. By validating the region features and pixel changes between the fractal deviations is defined as the addition of segmentation and detection rate of all features in such region is ∃


$$\rho {\left(C_{x}\right)}_{\exists }=\underset{―}{{\left({Sq}^{i}(r)\right)}^{2}}*{\left(D^{R}\right)}_{i}=\underset{―}{{\left({Sq}^{i}(r)\right)}^{2}}\sum_{n=0}{rf}_{n}^{i}*{pc}_{in}^{r}$$ (6)

**Fig 3:**
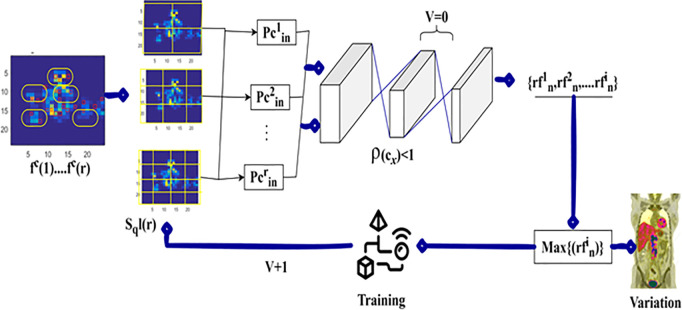
Variation due to detected region and maximum estimation.

In equation (6), the variable ${rf}_{n}^{i}$ determines the region features observed from the input image for addressing variations in any region based on pc for making better decisions to provide the diagnosis. If $\;{pc}_{in}^{r}$ represents the pixel changes based on variation detection observed at different regions. For example, $\;{pc}_{in}^{r}=0.07$ shows up the region detection precision with processing time and overhead, the number of pixel changes per unit of time is equal to 7% of the number of $\;F^{dv}\left(T^{SGM}\right)$ observed from the instance is estimated. The variation due to the detected region and its maximum estimation is presented in Fig 3.

The variation detection process utilizes $\;f^{C}(1)$ to $\;f^{c}(r)$ for $\;sq^{l}(r)$ validation. This validation requires  V=0 and $\;\rho \left(C_{x}\right)<1$ constraint sanctification such that the failing case generates $\;\left\{{rf}^{1}n\;to\;rf^{i}n\right\}$. This highlights the pixels with variations successively. In the contrary process, the $\left\{rf_{n}^{i}\right\}\;$ variation reflecting pixel is detected. This is used for training $\;Sq^{l}(r)$ through  (v+1)until  V=0 post the next sequence. The process is pursued until the variation is suppressed under $\;\rho \left(c_{x}\right)<1$ and  V=0 conditions (Fig 3).

### 3.3 Pixel changes in identified region

Based on the pixel changes, the problem location is identified, and the fractal deviations and feature changes are made using Swin Transformer to adapt the correlation. It is too difficult to find the precise region where the lymph node size may vary for all the patients, and providing a diagnosis also changes. In particular, the two conditions are followed to provide an appropriate diagnosis for lymphoma cancer: First, analyze the image, verify if the variation is happening, and help the affected patients with good treatment. Second, if maximum deviation is observed in any lymph node between the identified regions outputs high overhead and processing time. For instance, prescriptions are provided to the infected patients, and frequently going to the nearby medical centre for continuous health checks is the better solution for reducing lymphoma growth.

In this model, region detection and segmentation are performed to identify precise regions based on lymph node size and fractal deviations that may or may not correlate with the segmentation model. The transformer network trains feature changes and fractal deviations to address complex conditions in lymphoma detection based on PET images in different instances. The design goal of this model is to improve the detection accuracy and precision with less overhead. The training of the matching transformer network is terminated until a new change deviation observation or both observations. The proposed APS model formulation is estimated as


$${Max}_{{\left(D^{R}\right)}_{i}}=\sum pc\;\;\sum i\;\;\sum T\;\;{rf}_{n}^{\;\;i}*{pc}_{in}^{r}$$ (7)

Such that


$${pc}_{in}^{r}\leq {rf}_{n}^{\;\;i},\;if\;\forall \;i\in {\left({SGM}^{R}\right)}_{i},\forall n\in L_{r_{d}},\forall \;V$$ (8)

$$\begin{array}{c} \sum i\in L_{r_{d}}\;\;{pc}_{in}^{r}\leq 1,\;if\;\forall i\in \max_{dvt},\forall \;V \end{array}$$ (9)

$$\begin{array}{c} \sum i\;\;\max_{dvt}={\left({SGM}^{R}\right)}_{i} \end{array}$$ (10)

$${\Delta T}^{r}\leq \left(1-{\left(F^{dv}\left(T^{SGM}\right)\right)}_{r}\right)\left(1-f^{\;\;c}(r)\right),\;\forall r$$ (11)

$$\begin{array}{c} {rf}_{n}^{\;\;i};{pc}_{in}^{r}\in \left\{0,1\right\},\;if\;\forall i\in \max_{dvt},\forall n\in L_{r_{d}} \end{array}$$ (12)

Based on the above equations, the condition ${rf}_{n}^{\;\;i}=1$ is defined for training the ST network for detection and segmentation sequentially for addressing the lymphoma disease. If $L_{r_{d}}$ denotes the lymphoma region detection. The different prognosis of lymphoma cancer is improved using the proposed model and ST based on pixel changes and region features in any region. The condition $\;{pc}_{in}^{r}=1$ represents the variations addressed at n sequences for all the images. The region segmentation and detection time are computed for maximum fractal deviation x and changes identification y observation at the infection-located region for providing precise diagnosis. The pixel changes detection process in the identified region using Swin Transformer is presented below.

**Fig 4:**
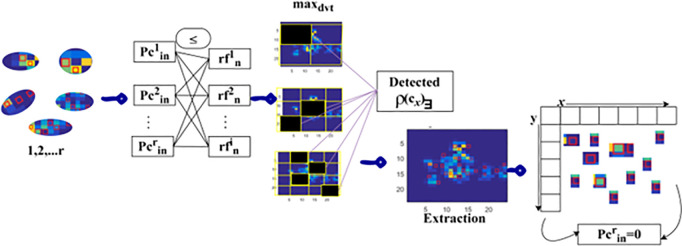
Pixel changes detection in *r.*

The inputs  r are observed for  V=0 and $\;\rho \left(C_{x}\right)<\;1-$pixel features from the $\;f^{c}(r)$. Depending on the condition $\;pc_{in}^{r}\leq rf_{n}^{i}$ the $\;m{ax}_{dit}$ between the successive pixels is identified. In this case, the condition failing pixels is identified as $\;\rho {\left(C_{x}\right)}_{\exists }$ from which the extraction is performed for  n. In this extraction, the $\;pc_{in}^{r}=0$ observed pixels are identified as the changes in any  r. Such changes are required for meeting $\;rf_{n}^{i};pc_{in}^{r}\in \left\{0,1\right\}$ suppressing further complexities (Fig 4). The maximum changes based on variation detection pseudo code are presented in Algorithm 2.

Algorithm 2: $\max_{dvt}$ Detection

$Input:r,\;C_{x}$

$Output:m{ax}_{dvt}$

Whilw n≠i loop condition

$\rho {\left(C_{x}\right)}_{\exists }=rf_{n}^{i}*pc_{in}^{r}$

$Initial:pc_{in}^{r}\leftarrow 1$

$If\;\left\{F^{dv}\leq \rho \left(C_{x}\right)*r\;\right\}then\;condition$

$pc_{in}^{r}=\frac{SGM^{R}}{r}*\frac{\rho \left(C_{x}\right)}{n}\;//Update$

$rf_{n}^{i}\leftarrow \{0,1\}$

$If\left\{rf_{n}^{i}=1\right\}then\;condition$

$r\leftarrow L_{r_{d}};\;\Delta T^{r}=\left[1-f^{c}(r)\right]\;//Update$

$Return\;m{ax}_{dvt}=0$

End If

End If

$If\;\left\{pc_{in}^{r}\leq rf_{n}^{i}\right\}then\;condition$

$\max_{dvt}=\left(SGM^{R}\right).\frac{i}{r}\;$

$C_{x}\leftarrow \frac{(DR{)}_{i}}{r*n}$

$Sq^{l}=V+1;\;\Delta T^{r}=\left[1-\frac{f^{\;\;c}(r)}{AV^{\theta }}\right]$

End If

End If

End while

### 3.4 Swin transformer in region detection

A hierarchical vision transformer called the Swin Transformer, which uses a shifted windowing method, may be very useful in an automated lymphoma pre-segmentation model that uses PET/CT data. To manage high-resolution vision tasks, the Swin Transformer (Shifted Window Transformer) partitions the input picture into non-overlapping local windows and calculates self-attention in each window. The shifted window technique provides cross-window linkages to improve the model’s computational efficiency and capacity to capture global context. Gather exquisite, co-registered CT and PET scans. The intensity levels should be normalized. resize photos into manageable patches that meet the input specifications of the Swin Transformer and resize them to good quality. To approximate where the lymphoma is located, thresholding or region expansion is utilized, using two classic segmentation approaches. The model’s starting point or preliminary guidance. Segment the PET/CT scans inputted as patches and insert them into vectors. The PET/CT scans extract multiscale and multidimensional characteristics using the Swin Transformer as a foundation. For better boundary accuracy and to deal with class imbalance, use a mix of segmentation-specific loss functions like Dice loss and cross-entropy loss in your model. Smooth down the segmentation mask’s edges and fine-tune it using operations like erosion and dilation. Determine how well the segmentation worked by calculating several metrics such as the Jaccard index, precision, recall, and F1-score. Swin Transformer’s hierarchical structure is essential to detect lymphoma areas that differ substantially in size and shape since it collects parameters at several scales. The automatic pre-segmentation of lymphoma in PET/CT images is made possible by Swin Transformer’s computational efficiency, global context awareness, and strong feature extraction capabilities. Better lymphoma diagnosis and treatment planning are made possible by this method’s increased accuracy and robustness in lymphoma segmentation.

From the instance, the feature extraction, false rate, and overhead are sequentially computed based on $\;\left(\max_{dvt}\times {\Delta T}^{r}\right)$ using ST for lymphoma region segmentation and detection. The probability of pixel changes $\;\left(\rho_{pc}\right)$ is computed sequentially and is given as


$$\rho_{pc}={\left(L_{r_{d}}+\max_{dvt}+{\Delta T}^{r}-1\right)}^{n-1}\;$$ (13)

Where


$$\rho_{L_{r_{d}}}=\left(1-\frac{{rf}_{n}^{i}\in \max_{dvt}}{{rf}_{n}^{i}\in {\Delta T}^{r}}\right)$$ (14)

As per equations (13) and (14), the continuous lymphoma region segmentation and detection is pursued based on observing the maximum deviation and changes for adapting the correlation between testing and training inputs. Therefore, there is less false rate observed from the instance and hence the precise detection is made for both conditions. Based on the region segmentation and detection, the new deviation or change is observed from $\;\rho_{L_{r_{d}}}$ and the precision of region detection is computed as


$$\rho_{pc}\left({\Delta T}^{r}\right)=\frac{1}{\left|L_{r_{d}}+\max_{dvt}+{\Delta T}^{r}-1\right|}\;.{\left(\rho_{L_{r_{d}}}\right)}_{in}\;$$ (15)

In equation (15), the high precision detection of lymphoma region is to satisfy minimum deviation and less overhead is valid for identifying segmentation changes. Based on the fractal deviations, the variation is identified due to metabolism effects across multiple tissues and lymph nodes. The multiple lymph nodes and tissues observed from the current metabolism measure used for identifying variations and overhead are reduced through the Swin Transformer network. The input segmentation uses extracted region features and pixel changes comparing the transformer network to prevent false rates provided to lymphoma-affected patients. The high accuracy and precision of region detection help to identify infected regions to achieve better output. Hence, the root cause and pixel changes are identified to achieve successful PET image processing with less overhead. Contrarily, the final region segmentation and detection output used for maximum deviation and changes identification is the optimal condition, and therefore, the segmentation time, processing time, and detection time for lymphoma based on PET/CT image processing results in a false rate. Therefore, the maximum deviation observed between the identified regions is used to improve detection precision and reduce the false rate. The lymph node is continuously segmented until it identifies the maximum deviation and changes. Here, the segmentation and detection rate are used to achieve decision-making and better output. Therefore, the false rate identified pixel changes when matching the transformer network with the final output to provide optimal diagnosis to the affected people.

## 4. Experimental analysis

The experimental analysis presents the discussion using MATLAB outputs image inputs [34] and proposed models’ correlation. First, the data source is explained; whole-body PET/CT Scan image inputs are used for experimental analysis. This dataset uses 501 input images obtained at 200mAs reference dosage at 120kV tube voltage post 60 mins of IV. The image sizes are consecutively 1x1, 2x2, 4x4, 8x8, and 16x16, extracted from a larger image of 400x400 sizes. From the model’s correlation, the output obtained and its statistical analysis is discussed below.

### 4.1. Analysis-I: variation and regions

**Fig 5:**
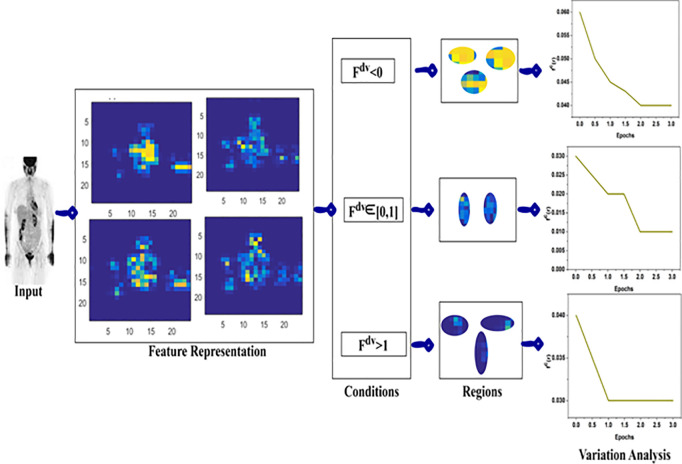
Variation and region analysis.

The optimal condition for deviation is $\;F^{dv}\in [0,1]$ shows up less variation under three epochs. This requires a high convergence for $\;F^{dv}<0$ and low for $\;F^{dv}>1$. Therefore the number of epochs and training recurrences are decided from $\;C_{x}\forall f^{c}(r)$ Fig 5. Followed by this Analysis II presents the region detection under $\;m{ax}_{dvt}$.

### 4.2. Analysis-II: region detection based on $\;m{ax}_{dvt}$

**Fig 6:**
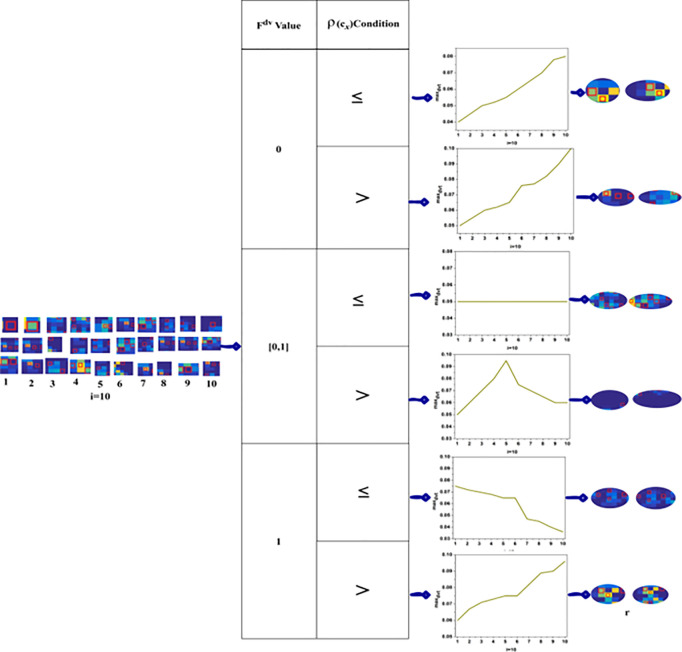
$m{ax}_{dvt}$-based Regions.

The $\;max_{dvt}$ region detection is performed using $\;F^{dv}$ and $\;\rho \left(c_{x}\right)$ inputs combined using ≤or> conditions. Therefore for $\;F^{dv}$ and $\;\rho \left(C_{x}\right)$, $\;6\;max_{dvt}$ the analysis is presented in the above Fig 6. This is performed for 10  i(i<n) from the original input. The experimental outcomes are tabulated and graphically represented for $max_{dvt}$ in the above. The regions represented above are the $\;max_{dvt}$ expressing ones that are distinguished from the precision region. The final precision region and its best error are presented in Analysis III.

### 4.3. Analysis-III: precision region detection

**Fig 7:**
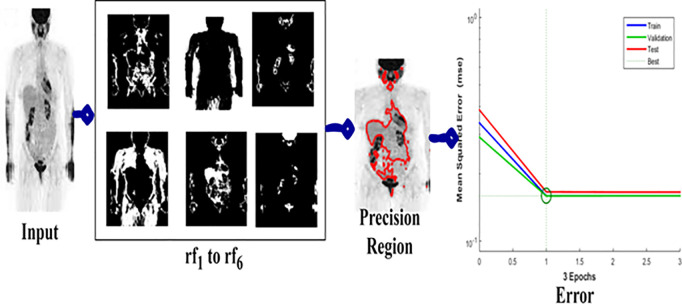
Precision region detection.

In Fig 7 above, the region detected by satisfying the conditions presented in Fig 6 is presented. For example, the consideration is about 6 regions identified for which the training, validation, and testing outputs for 3 epochs are analyzed. In this representation process, the best solution and convergence are cumulatively identified unlike the conditional analysis and regions presented. The precision region exhibits some changes in detecting lymphoma that is different from the deviation observed regions.

## 5. Results, discussion and comparative analysis

The comparative analysis uses segmentation accuracy, precision, overhead, error, and segmentation time metrics. The features (1–14) and regions (5) are varied for analyzing the metrics above. For an effective comparative analysis, the existing AW-SDRLSE [19], DFE + ES [24], and PSR-Nets [26] methods are considered along the proposed APSM-ST.

The current approaches often provide PET pictures with inferior spatial resolution and greater noise levels when compared with CT scans. Lack of proper integration might result in subpar performance in feature extraction and segmentation, preventing the advantages of multimodal imaging from being fully realized. A model’s clinical value is limited if it does well on the training set but fails to segregate lymphoma in fresh patient data correctly. Hence this paper compared AW-SDRLSE [19], DFE + ES [24], and PSR-Nets [26] and this article analyses the lymphoma segmentation using PET/CT images.

### 5.1. Segmentation accuracy

**Fig 8:**
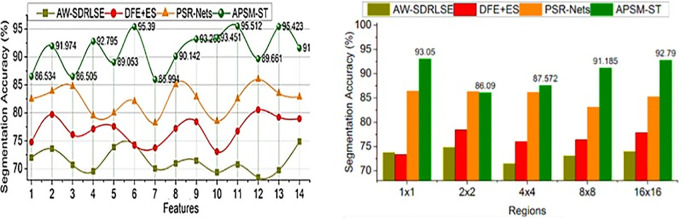
Segmentation accuracy for features and regions.

In this proposed model, the Swin Transformer is used for performing continuous region segmentation and detection process for improving the diagnosis rate and lymphoma detection rate based on variation analysis shown in Fig 8.

The error occurred due to high metabolism effects addressed across different tissues and lymph nodes observed from the input PET images to achieve high segmentation accuracy. The identified variations are mitigated using the APSM and transformer network to reduce the growth of lymph node size for disease-causing intervals. The extracted features from PET/CT images are sequentially processed to achieve maximum detection precision with less overhead and error. The consecutive region features and pixel changes are trained based on the fractal deviations to reduce error occurrence. To address the above complex problems, the proposed model is used to identify lymphoma disease-infected regions based on the maximum deviation and changes. This achieves high segmentation accuracy at the time of identifying the variations between the regions that are confined unanimously through the proposed model. The segmentation accuracy is improved by 12.68% and 13.75% for the varying features and sizes, respectively.

### 5.2. Precision

**Fig 9:**
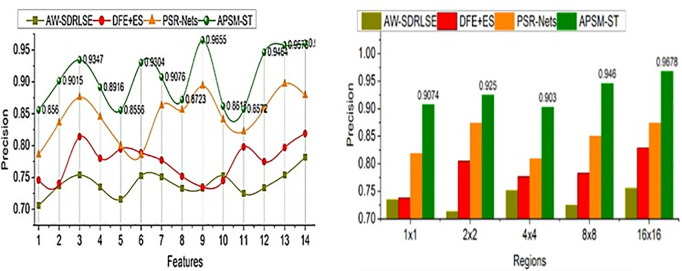
Precision for features and regions.

In this proposed model using ST is to satisfy high region detection precision based on the pixel changes identified across various regions in any time interval represented as in Fig 9. The minimum or maximum variations are detected from the extracted features using Swin Transformer to improve the segmentation rate. The pixel changes and region features are accurately identified for lymphoma region segmentation based on the deviation identification. In this article, the ST network trains feature changes and fractal deviations are processed unanimously to adapt the correlation between testing and training inputs at different instances. The APSM is used to identify lymphoma disease causing reason and controlling the infection spread based on feature extraction. The maximum fractal deviation observed between the lymphoma-detected regions is pursued using the proposed model at any region. The proposed model’s conditions are computed to satisfy high segmentation accuracy and detection precision. The detection precision is high compared to the other factors in this model. The precision is hiked by 13.38% and 14.81% for the features and sizes of the proposed APSM.

### 5.3. Overhead

**Fig 10:**
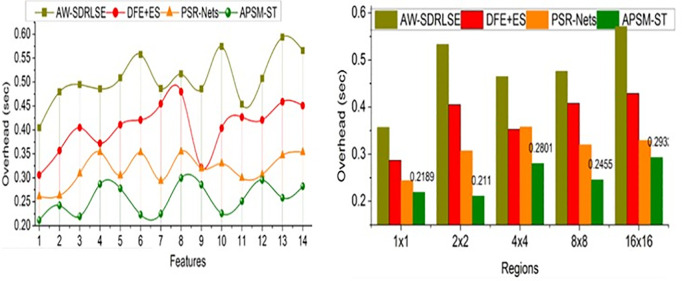
Overhead for features and regions.

The proposed model used Swin Transformer for lymphoma-affected region detection precision to satisfy a high segmentation rate with less overhead for preventing the error occurrence at any region shown in Fig 10. Identifying variations observed from the extracted features leads to high error occurrence; the considerable features are changed to appropriate pixel attributes using the transformer network. Therefore, the lymph node size is accurately identified to provide appropriate diagnosis recommendations. Based on the maximum and minimum deviations, the pixel changes are classified to detect and segment lymphoma regions in the input PET/CT images. The proposed model first segments the input in this model based on pixel changes and variations to improve the accuracy of segmentation and detection rate. By validating the precise segmentation and region detection based on maximum deviation and pixel changes observed from the PET/CT images. The precise region detection is difficult to meet, and the maximum deviation relies on the lymph node size and fractal deviations using the Swin Transformer network. The features extracted from given input images are processed in any region at different time intervals. The proposed model is used to satisfy less overhead. Overhead is reduced by 12.73% and 11.27% by the proposed model for different features and sizes.

### 5.4. Error

**Fig 11:**
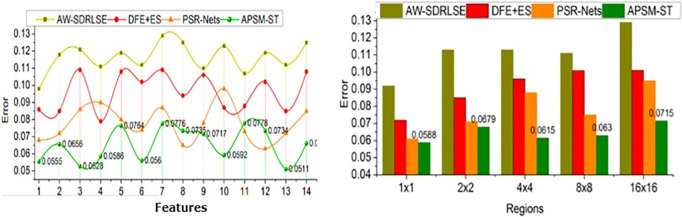
Error for features and regions.

In this proposed model, the maximum deviation is addressed concerning the sequence of segmentation changes and region features to improve the detection rate for better decision-making shown in Fig 11.

The continuous training of the Swin Transformer network is pursued for precise region segmentation, and detection is performed simultaneously to adapt the correlation. The pixel changes may differ for each lymph node based on extracted feature attributes; the observed image is processed using ST to make accurate and appropriate diagnosis recommendations. In this article, the training of the matching transformer network is terminated until a new change or deviation from the instance across various regions is to prevent errors and overhead. In this scenario, segmentation and detection are performed to reduce errors and overheads and maximize the precision of region detection. The maximum/minimum fractal deviations are identified and segregated from the training inputs and testing to improve the segmentation accuracy. Instead, the variation analysis and minimum deviation control the lymphoma disease. Hence, fewer errors are satisfied using this model and ST. The proposed APSM reduces error by 9.27% for the features and 11.05% for the sizes compared to the other methods.

### 5.5. Segmentation time

**Fig 12:**
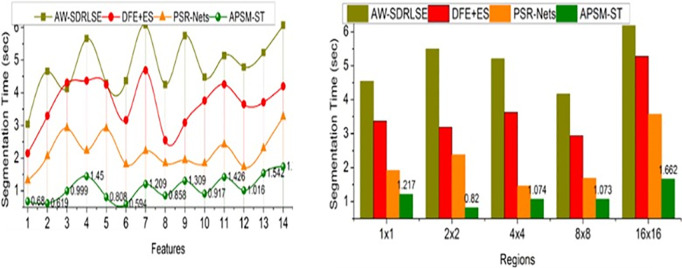
Segmentation time for features and regions.

This proposed model aided in better decision-making of lymphoma region detection without errors and overheads to satisfy less detection time and thereby reduce detection time compared to the other factors represented in Fig 12. The variations are identified from the disease-infected regions to reduce maximum deviations and changes between the region features and pixel changes are observed to improve detection precision with less overhead. This proposed model identifies the complex problems observed from sequential image processing. The abovementioned variations are addressed using ST based on the fractal deviations and feature changes. The training of the same transformer network is terminated using the proposed model to perform precise region segmentation and detection. The proposed model notices the frequency fluctuations in its associated features to reduce errors and overheads. The above problems are difficult to identify; based on the extracted features regions and changes, additional features are added to reduce the maximum deviation. Therefore, the proposed model satisfies less segmentation time. As Compared to the other methods, the segmentation time is reduced by 10.23% and 11.14% under varying features and sizes.

### 5.6 Confusion matrix

The visualization of the confusion matrix draws attention to the superior segmentation performance of the APSM-ST model that was developed. Not only does it have the highest true positive (780) and true negative (1100) numbers, but it also has the fewest incorrect classifications (20 false positives and 45 false negatives), which indicates that it has exceptional precision and recall. The APSM-ST consistently minimises the number of misclassifications while maintaining balanced detection across object and background classes. This is in comparison to other models such as PSR-Nets, DFE + ES, and AW-SDRLSE. This not only strengthens the model’s robustness and dependability in the process of segmenting complex picture regions, but it also makes it a more effective solution for high-accuracy image segmentation jobs in real-time applications.

**Fig 13:**
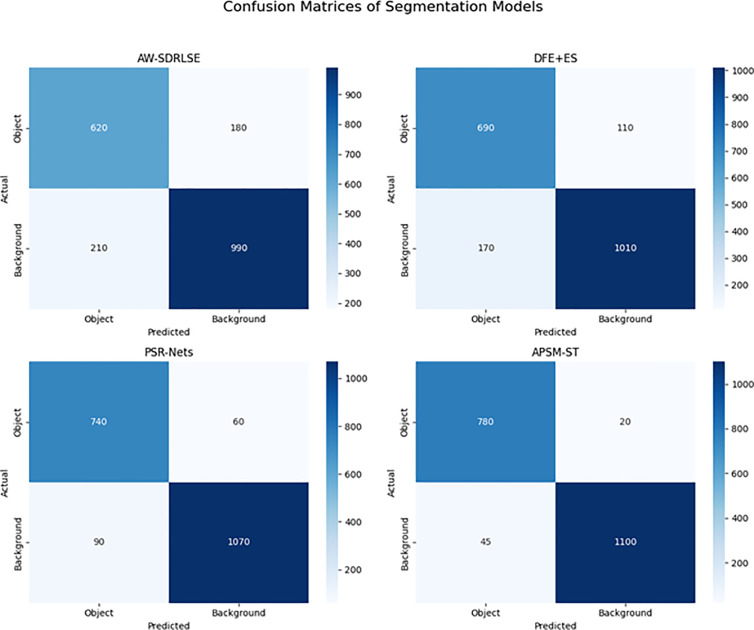
Confusion matrix for segmentation models.

In the comparison Table 1, the performance of four different segmentation models—AW-SDRLSE, DFE + ES, PSR-Nets, and the proposed APSM-ST—is illustrated across many assessment parameters. These metrics include segmentation accuracy, precision, computational overhead, error rate, and segmentation duration. The APSM-ST algorithm consistently achieves superior results across all metrics and region sizes. It achieves the highest accuracy (up to 93.05%), maximum precision (0.9678), lowest overhead (0.2189 seconds), minimal error (0.0588), and the quickest processing time (1.217 seconds). These findings support the resilience of the proposed model and demonstrate that it is suitable for accurate and efficient medical image segmentation Fig 13. Furthermore, they show the potential of the model for real-time applications that have high reliability and little resource utilisation.

**Table 1:** Comparative performance analysis of segmentation models across various metrics.

Metric	Region	AW-SDRLSE	DFE + ES	PSR-Nets	APSM-ST (Proposed)
**Accuracy (%)**	1 × 1	72.5	76.3	86.5	**93.05**
	2 × 2	73.1	77.8	83.7	**86.09**
	4 × 4	72.4	75.9	87.572	**87.572**
	8 × 8	73.6	75.1	88.7	**91.185**
	16 × 16	74.8	76.4	89.9	**92.79**
**Precision**	1 × 1	0.731	0.782	0.856	**0.9074**
	2 × 2	0.75	0.793	0.872	**0.925**
	4 × 4	0.738	0.779	0.903	**0.903**
	8 × 8	0.747	0.785	0.915	**0.946**
	16 × 16	0.762	0.802	0.926	**0.9678**
**Overhead (sec)**	1 × 1	0.36	0.29	0.27	**0.2189**
	2 × 2	0.58	0.41	0.33	**0.211**
	4 × 4	0.52	0.39	0.31	**0.2801**
	8 × 8	0.54	0.42	0.28	**0.2455**
	16 × 16	0.6	0.46	0.33	**0.293**
**Error**	1 × 1	0.11	0.081	0.0656	**0.0588**
	2 × 2	0.12	0.094	0.0679	**0.0679**
	4 × 4	0.12	0.087	0.0764	**0.0615**
	8 × 8	0.115	0.098	0.0715	**0.063**
	16 × 16	0.13	0.099	0.0734	**0.0715**
**Seg. Time (sec)**	1 × 1	4.1	2.8	2.1	**1.217**
	2 × 2	5.6	3.2	2.4	**0.82**
	4 × 4	4.3	2.9	2.3	**1.074**
	8 × 8	4.5	3.1	2.4	**1.073**
	16 × 16	6.1	3.5	2.8	**1.662**

## 6. Conclusion

This study presents an automated pre-segmentation model to improve the precision of identifying lymphoma in PET/CT scans. Using a Swin Transformer, the model can segment and detect lymphoma in whole-body PET image analysis. It accomplishes accurate detection of pixel changes that lack increasing complexity or generate false positives, guaranteeing great precision in region detection which is important for managing lymphoma. The methodology uses a step-by-step process of decision-making and calculation to fight against lymphoma sickness effectively. Teaching the matching transformer network allows for effective region detection, with completed examples of the network recognized under different input elements. The model correctly identifies lymphoma disease in various areas by considering differences in range characteristics and pixels detected during PET image analysis. The comparison shows notable enhancements: segmentation accuracy rises by 12.68% and precision by 13.38%. Moreover, the model decreases overhead, error rates, and division time by 12.73%, 9.27%, and 10.23% respectively for various features.

## 7. Future works

However, initial segmentation procedures, such as categorization and detection, are influenced by differences in lymph node diameters. Differences in mistakes occur due to the different widths of areas, affecting the distribution of pixels in sequence. A method called regressive segmentation is suggested to address this problem. This method examines pixel distribution separately according to the size of the region. This method seeks to tackle issues related to pre-segmentation to improve the accuracy of lymphoma diagnosis in PET/CT images.
